# Pathways to HIV testing and care in Goa, India: exploring psychosocial barriers and facilitators using mixed methods

**DOI:** 10.1186/s12889-016-3456-4

**Published:** 2016-08-11

**Authors:** Rosie Mayston, Anisha Lazarus, Vikram Patel, Melanie Abas, Priya Korgaonkar, Ramesh Paranjape, Savio Rodrigues, Martin Prince

**Affiliations:** 1 Health Service & Population Research Department, Kings College London, London, SE5 8AF UK; 2 Centre for Global Mental Health, London School of Hygiene & Tropical Medicine, London, WC1E 7HT UK; 3 Sangath, H No 451 (168), Bhatkar Waddo, Socorro, Porvorim, Bardez, Goa 403501 India; 4 National AIDS Research Institute, 73, ‘G’-Block, MIDC, Bhosari, Pune 411 026 India; 5 Department of Microbiology, Goa Medical College and Hospital, Bambolim, Tiswadi, Goa India

## Abstract

**Background:**

Despite recognition of the importance of timely presentation to HIV care, research on pathways to care is lacking. The adverse impact of depression upon adherence to antiretroviral therapy is established. There is emerging evidence to suggest depression may inhibit initial engagement with care. However, the effect of depression and other psychosocial factors upon the pathway to care is unknown.

**Methods:**

We used mixed methods to explore pathways to care of people accessing testing and treatment in Goa, India. Questionnaires including measures of common mental disorder, hazardous alcohol use, cognition and assessment of pathways to care (motivations for testing, time since they were first aware of this reason for testing, whether they had been advised to test, who had given this advice, time elapsed since this advice was given) were administered to 1934 participants at the time of HIV testing. Qualitative interviews were carried out with 15 study participants who attended the antiretroviral therapy treatment centre. Interview topic guides were designed to elicit responses that discussed barriers and facilitators of accessing testing and care.

**Results:**

Pathways were often long and complex. Quantitative findings revealed that Common Mental Disorder was associated with delayed testing when advised by a Doctor (the most common pathway to testing) (AOR = 6.18, 2.16–17.70). Qualitative results showed that triggers for testing (symptoms believed to be due to HIV, and for women, illness or death of their husband) suggested that poor health, rather than awareness of risk was a key stimulus for testing. The period immediately before and after diagnosis was characterised by distress and fear. Stigma was a prominent backdrop to narratives. However, once participants had made contact with care and support (HIV services and non-governmental organisations), these systems were often effective in alleviating fear and promoting confidence in treatment and self-efficacy.

**Conclusion:**

The effectiveness of formal and informal systems of support around the time of diagnosis in supporting people with mental disorder is unclear. Ways of enhancing these systems should be explored, with the aim of achieving timely presentation at HIV care for all those diagnosed with the disease.

## Background

Early diagnosis and timely treatment can enable people diagnosed with HIV to live long lives lived largely free from HIV-related morbidity [1, 2]. Late presentation to care, defined as presenting with a CD4 count of less than 350 cells/ml or attending care with an AIDS-defining illness [3], is associated with poor outcomes for individual patients (increased risk of morbidity, death) as well for treatment programmes (increased costs) [4]. Although CD4 counts at the time of initiating Antiretroviral Therapy (ART) have improved over time within many individual programmes, a recent systematic review of aggregated data from African studies showed no change in CD4 counts between 2002 and 2013 [5]. Ensuring pathways to testing and care are short and direct, ie. testing and diagnosis prior to the onset of HIV/AIDS-related illness, presentation at HIV care promptly after diagnosis, and ART initiated as soon as eligibility criteria are met, is an important component of working towards an AIDS-free generation [6].

Although studies looking at pathways to care are common in psychiatry [7, 8], we were unable to identify any studies that examined pathways to HIV testing and care. Despite the body of evidence regarding the risks associated with late presentation, our understanding of why people might delay testing for HIV and/or linkage to care until they are very sick is limited. Although innovations in HIV services, such as provider-initiated testing, provision within primary care etc. have reduced systems level barriers to testing and treatment, service-level barriers cannot be completely eliminated [9–11]. Likewise, economic constraints, associated with the prioritisation of basic needs for food, accommodation, clothing over healthcare [12] have been suggested as potentially important barriers to timely testing and care [13]. Stigma has been identified as an important deterrent to timely testing and treatment uptake [14, 15].

Depression and heavy alcohol use are known to be associated morbidity and mortality and sub-optimal adherence to ART [16–19]. The role of alcohol in determining pathways to care and timing of presentation to care is unclear [20, 21]. A recent study in South Africa found that those with poor emotional health were more likely to be late presenters [12]. In our study in Goa (Umeed), we found that those with depressive symptoms were less likely to collect HIV test results and less likely to attend care [22]. However, the role of common psychological morbidities and other psychosocial factors in the earlier stages of the continuum of care (pathways to testing, initiating contact with Antiretroviral Therapy (ART) services) is largely unexplored.

In order to understand how these and other factors might contribute to late presentation, it is necessary to better understand peoples’ pathways from before they undergo HIV testing, through to receiving their diagnosis, initiating care and starting ART.

## Methods

### Design

Findings presented here come from analysis of baseline quantitative and nested qualitative data from the Umeed cohort study carried out in Goa, India [22–24]. The overall aim of Umeed was to investigate the impact of common mental disorders, hazardous alcohol use and cognitive impairment upon access to HIV testing and care. The Umeed cohort consisted of 1934 participants, recruited at the time of coming for HIV testing and followed up for a minimum of 1 month to ascertain whether those who tested positive had linked to HIV care. We analysed baseline quantitative data and qualitative interviews with a purposive sample of HIV-positive participants accessing treatment and care for HIV to address the following aims.

### Study aims

- To describe the pathways to care of cohort participants
- to examine the role of common mental disorder, hazardous alcohol use and cognitive impairment in determining pathways
- to explore the role of other psychosocial factors in shaping the experience of accessing HIV testing and care.

### Setting

Goa is an Indian state with a population of 1.34 million (Government of Goa 2010, https://www.goa.gov.in/knowgoa/aboutgoa.html, accessed 23 September 2010). For the purposes of HIV/AIDS surveillance, Goa is divided into two districts: at the time of data collection, the north was one of India’s high prevalence districts, with more than one percent prevalence among women attending for antenatal care. The south was medium prevalence, with a high prevalence among high-risk groups (more than five percent among those attending STI clinics) (United Nations General Assembly Special Session 2008, http://data.unaids.org/pub/Report/2008/india_2008_country_progress_report_en.pdf, accessed 23 May 2010). The study was located at Goa Medical College (GMC), the only tertiary level hospital in the state and home to the sole public ART Treatment Centre in Goa. GMC is centrally located on a main road and bus routes just outside of the state capital, Panjim.

### Participants

The cohort was recruited from attendees of the Integrated Counselling and Testing Centre (ICTC). Participants were recruited between January 2008 and January 2010 and followed-up until March 2010, at the time of attending the ICTC for pre-test counselling and testing. Participants were either: “walk-in” clients (self-referrals, or primary care physician referrals) or those formally referred for testing by doctors from other GMC departments. Those attending for the first time, for pre-test counselling and a HIV blood test, and who spoke Konkani, Hindi, or English were eligible to participate. Participants were eligible for the qualitative study if they had: a) collected test results and attended the ART Centre; b) agreed to be approached after collection of test results about participation in the qualitative study (at the time of consent to participate in the quantitative study). Researchers asked attendees of the ART Centre whether they had participated in the quantitative study. Participants were asked for their Patient Identification (PID) number (unique identifier assigned by ICTC Counsellors). This was linked to study identification numbers. Those participants who had agreed to being approached were provided with further information about the qualitative study and asked to sign a second consent form, using the same procedures as at baseline. Purposive sampling was carried out to select participants with a range of characteristics that were expected to affect their experience of testing and accessing services for HIV/AIDS (gender, time since diagnosis, symptoms of Common Mental Disorder (CMD), Alcohol Use Disorder (AUD) and cognitive impairment).

### Quantitative measures

Questionnaires were administered by research assistants to participants at the time of attendance for HIV-testing. Questions relating to sociodemographic characteristics, HIV-related behaviours, beliefs and knowledge (transmission and prevention knowledge, disclosure plans, sexual behaviour, knowledge and symptoms of sexually transmitted infections) and a screening assessment for mental, substance and alcohol use disorders and cognitive testing were included. Basic sociodemographic data was collected from those who declined to participate.

CMD was measured using the nine item Patient Health Questionnaire (PHQ-9), the seven item Generalised Anxiety Disorder scale (GAD-7), and the panic disorder module [25]. The PHQ-9 has been previously validated in Goa, for the detection of CMD in primary care, being one of five instruments to achieve an area under the curve (AUC) from receiver operating characteristic analysis of at least 0 · 80 (0 · 84 for the PHQ-9) [26]. The 10-item Alcohol Use Disorder Identification Test (AUDIT) was used to screen for hazardous and dependent alcohol use [27]. The instrument has been widely used in India [28] and in Goa [29]. Two measures of cognitive functioning were included: word list learning (assessing memory) and animal naming (measuring verbal fluency, an aspect of executive functioning, known to be a common deficit in people living with HIV/AIDS [30]. A full description of questionnaires, including mental health and cognition measures can be found elsewhere in our open access paper [23].

Pathways to care: Given the lack of literature on pathways to HIV care at the time of study design, our pathways to care questionnaire was devised specifically for use in this study, with reference to the pathways to psychiatric care literature (eg. [31]). Participants were asked to indicate their motivations for testing from a list of options developed from formative qualitative interviews with local non-governmental organisation (NGO) and ICTC, ART Centre staff. Options included in the final questionnaire were: pre-marital testing; test in preparation for working abroad; having a child or partner who was HIV-positive; partner’s risk behaviour; symptoms believed to be due to HIV; risk behaviour. For each of the motivations indicated, participants were asked to estimate the time elapsed since they first became aware of this reason for testing (less than 1 week; more than a week but less than a month; more than a month but less than a year; more than a year). Next, participants were asked to identify any persons who had advised them to undergo testing for HIV (ie. Doctor, NGO staff member, family member, friend) and estimate the length of time elapsed since this advice was received (less than 1 week; more than a week but less than a month; more than a month but less than a year; more than a year).

### Qualitative interviews

Fifteen interviews were carried out in local languages by two Research Assistants trained in social science and with experience of qualitative interviewing. Topic guides for interviews were developed with the aim of investigating psychosocial barriers and facilitators of HIV testing and accessing care for HIV/AIDS. Participants were asked to reflect on their attitudes, thoughts, feelings and responses (actions) at different time-points in their pathway to the ART Centre. Interviews began by exploring the period before testing. Participants were asked about their motivations for testing and factors that had encouraged or discouraged testing. Interviews examined experiences of pre-test counselling and testing and post-test counselling and attending the ART Centre, including interactions with clinic staff. Interviews lasted between 30 and 40 min and were carried out at the ART Centre or in private interview cabins at the ICTC (according to the preference of the participant).

### Data analysis

Our hypothesis was that CMD, AUD and cognitive impairment would be independently associated with longer pathways to care, ie. More time elapsed between a) first awareness of motivations for testing and attending the ICTC for testing b) being advised to test and attending the ICTC for testing. In the testing of both hypotheses, we moved from bivariate (chi-squared tests for homogeneity and crude odds ratios) to multivariable analyses (logistic regression) controlling for the effects of gender, education and age. Due to the negligible prevalence of AUD among women (0·9 %), all analyses of alcohol use were carried out using a men-only sample (*n* = 915).

We analysed the qualitative data using thematic analysis to develop a coding framework and identify emerging themes. Two of the researchers (RM, AL) read the first five qualitative interviews repeatedly to immerse themselves in the data. Using NVIVO software, RM and AL labelled meaningful pieces of text with appropriate codes and sub-codes. The two independent sets of codes were compared and amalgamated into a single framework. Emergent themes were discussed. RM applied the coding framework to the full dataset and reconciled the final themes.

## Results

### Quantitative

There was strong evidence to support an association between CMD and delaying testing to after 1 month of being advised by a Doctor and weaker evidence to suggest a similar association with being advised to test by an NGO staff member (AOR = 6.18, 95 % CI = 2.16–17.70; OR = 56.0, 95 % CI = 1.93–1.62e + 03, respectively) (Table 1). There was some evidence to suggest that CMD was associated with delaying coming to test after knowing about partner’s risk. In bivariate analyses, CMD was found to be inversely associated with reporting repeat testing (OR = 0.2, 95 % CI = 0.28–0.96). Although the magnitude of this association altered little after adjusting for gender, education and age, it was no longer statistically significant (AOR = 0.55, 95 % CI = 0.30–1.03). CMD was also associated with reporting risk behaviour as a reason for testing (AOR = 3.04, 95 % CI = 1.68–5.41). AUD was associated with not giving a reason for testing (AOR = 1.68, 95 % CI = 1.25–2.27) and reporting risk behaviour as a reason for testing (AOR = 1.95, 95 % CI = 1.16–3.27).

**Table 1:** Associations between reason for testing, length of time aware of risk, route to testing and CMD/AUD

Reason for testing, length of time aware of risk, route to testing	All	CMD	*P*-value (chi-squared)	OR (95 % CI)	^a^AOR (95 % CI)	^b^AUD	*P*-value (chi-squared)	OR (95 % CI)	^a^AOR (95 % CI)
No reason given	1095 (56.6)	51 (6.1)	0.23	1.27 (0.86–1.89)		133 (32.8)	<0.01	1.68 (1.2–2.26)	1.68 (1.25–2.27)
Pre-marital test	29 (1.5)	2 (6.9)	0.88	1.31 (0.31–5.58)		4 (20.0)	0.47	0.67 (0.22–2.02)	
Repeat test	378 (19.5)	12 (3.2)	0.03	0.52 (0.28–0.96)	0.55 (0.30–1.03)	57 (30.7)	0.22	1.24 (0.87–1.77)	
Test before going abroad	30 (1.6)	1 (3.3)	0.81	0.60 (0.08–4.47)		9 (34.6)	0.38	1.44 (0.63–3.28)	
Child/partner who is HIV + ve	119 (6.2)	7 (5.9)	0.80	1.11 (0.50–2.44)		8 (30.8)	0.67	1.20 (0.52–2.80)	
Known for <1 month	47 (39.5)	2 (4.3)				1 (16.7)			
Known for >1 month	72 (60.5)	5 (6.9)	0.54	1.68 (0.31–9.12)		7 (35.0)	0.11 (2.62)	4.95 (0.57–43.04)	
Partner’s risk behaviour	91 (4.7)	6 (6.6)	0.60	1.26 (0.54–2.95)		11 (39.3)	0.14	1.77 (0.82–3.85)	
Known for <1 month	37 (40.7)	0 (0.0)				4 (33.3)	4 (36.4)		
Known for >1 month	54 (59.3)	6 (11.1)	0.04	NA	NA	6 (37.5)	7 (63.6)	1.56 (0.32–7.64)	
Symptoms which you believe may be due to HIV	77 (4.0)	8 (10.4)	0.05	2.13 (0.99–4.55)	2.26 (1.03–4.96)	18 (34.6)	0.21	1.46 (0.81–2.63)	
Known for <1 month	47 (61.0)	2 (4.3)				14 (38.9)			
Known for >1 month	30 (39.0)	6 (20.0)	0.03	5.63 (0.99–32.12)	15.53 (1.53–157.68)	4 (25.0)	0.33	0.52 (0.14–2.00)	
Risk behaviour	112 (5.8)	15 (13.4)	<0.01	3.01 (1.68–5.41)	3.04 (1.67–5.55)	28 (41.8)	0.01	2.05 (1.23–3.42)	1.95 (1.16–3.27)
Known for <1 month	60 (53.6)	6 (10.0)				13 (35.1)			
Known for >1 month	52 (46.4)	9 (17.3)	0.26	1.88 (0.62–5.77)		15 (50.0)	0.22	1.85 (0.68–5.03	
Advised to test by Doctor	1457 (75.3)	84 (5.8)	0.19	1.40 (0.85–2.30)		166 (28.0)	0.44	1.13 (0.83–1.54	
<1 month ago	1433 (98.4)	79 (5.5)				164 (28.0)			
>1 month ago	24 (1.7)	5 (20.8)	<0.01	4.51 (1.63–2.44)	6.18 (2.16–17.70)	2 (22.2)	0.70	0.73 (0.15–3.57	
Advised to test by NGO staff	60 (3.1)	3 (5.0)	0.90	0.92 (0.28–3.00)		16 (?)			
<1 month ago	58 (95.1)	2 (3.5)				16 (6.6)			
>1 month ago	3 (4.9)	2 (66.7)	<0.01	56.00 (1.93–1.624e + 03)	NA	0 (0.0)	NA	NA	
Advised to test by family member/friend/other	71 (3.7)	1 (1.4)	0.13	0.24 (0.03–1.78)					
<1 month	47 (66.2)	1 (2.1)				3 (15.0)			
>1 month ago	24 (33.8)	1 (0.0)	0.47	NA		3 (21.4)	0.63	1.55 (0.25–9.38)	

^a^Adjusted for gender, education, age

^b^Analysed using a men-only dataset, *n* = 915

There was evidence to suggest that low score on the delayed recall test was inversely associated with delaying testing to after 1 month of being aware of symptoms that may be due to HIV (AOR = 0.22, 95 % CI = 0.05–0.96) (Table 2). Low score on the test of verbal fluency was inversely associated with delaying testing for more than 1 month after being aware of risk behaviour (AOR = 0.22, 95 % CI = 0.08–0.57). There was weak evidence that low score on the test of delayed recall was associated with delaying testing for more than 1 month after being advised by a family member, friend or person other than a Doctor/NGO worker (OR = 2.81, 95 % CI = 0.80–9.95; AOR = 2.68, 95 % CI = 0.69–10.42). Low score on the delayed recall cognitive test was inversely associated with testing before going abroad (AOR = 0.11, 95 % CI = 0.01–0.79). In bivariate analysis, there was weak evidence of an association between low score on the delayed recall test and having a HIV-positive partner or child (OR = 1.42, 95 % CI = 0.94–2.14); however, after adjusting for potential confounders, this association reached statistical significance (AOR = 1.31, 95 % CI = 1.06–1.62). Low score on the verbal fluency test was associated with having a child or partner who was living with HIV (AOR = 1.31, 95 % CI = 1.07–1.60).

**Table 2:** Associations between reason for testing, length of time aware of risk, route to testing and cognitive impairment (delayed recall and verbal fluency) among people attending for HIV testing in Goa, India (*n* = 1934)

Reason for testing	Low score- delayed recall	*P*-value (chi-squared)	OR (95 % CI)	^a^AOR (95 % CI)	Low score- verbal fluency	*P*-value (chi-squared)	OR (95 % CI)	^a^AOR (95 % CI)
No reason given	258 (23.6)	0.55	0.94 (0.76–1.16		280 (25.6)	0.79	1.03 (0.84–1.27	
Pre-marital test	4 (13.8)	0.36	0.53 (0.18–1.53)		6 (20.7)	0.57	0.75 (0.30–1.84)	
Repeat test	80 (21.2)	0.45	0.87 (0.66–1.15)		91 (24.1)	0.47	0.89 (0.68–1.16)	
Test before going abroad	1 (3.3)	0.01	0.11 (0.02–0.84)	0.11 (0.01–0.79)	7 (23.3)	0.56	0.87 (0.37–2.04)	
Length of time aware of risk								
Child/partner who is HIV + ve	35 (29.4)	0.09	1.42 (0.94–2.14)	1.31 (1.06–1.62)	42 (35.3)	0.02	1.62 (1.09–2.39)	1.31 (1.07–1.60)
Known for <1 month	14 (29.8)				14 (29.8)			
Known for >1 month	21 (29.2)	0.94	0.97 (0.43–2.18)		28 (38.9)	0.31	1.50 (0.68–3.31)	
Partner’s risk behaviour	26 (28.6)	0.20	1.36 (0.85–2.16)		27 (29.7)	0.39	1.22 (0.77–1.94)	
Known for <1 month	11 (29.7)				13 (35.1)			
Known for >1 month	15 (27.8)	0.84	0.91 (0.36–2.30)		14 (25.9)	0.35	0.65 (0.26–1.62)	
Symptoms which you believe may be due to HIV	18 (23.4)	0.95	1.02 (0.59–1.75)		21 (27.3)	0.77	1.08 (0.65–1.80)	
Known for <1 month	14 (29.8)				14 (29.8)			
Known for >1 month	4 (13.3)	0.10	0.36 (0.10–1.27)	0.22 (0.05–0.96)	7 (23.3)	0.54	0.72 (0.25–2.07)	
Risk behaviour	28 (25.0)	0.62	1.12 (0.72–1.74)		36 (32.1)	0.12	1.39 (0.92–2.09)	
Known for <1 month	19 (31.7)				26 (43.3)			
Known for >1 month	9 (17.3)	0.08	0.45 (0.18–1.13)	0.29 (0.10–0.83)	10 (19.2)	0.01	0.31 (0.13–0.76)	0.22 (0.08–0.57) P < 0.01
Route to testing								
Advised to test by Doctor	339 (23.3)	0.71	1.05 (0.82–1.34)		363 (24.9)	0.10	0.82 (0.65–1.04)	0.82 (0.64–1.04)
<1 month ago	334 (23.3)				358 (25.0)	0.24		
>1 month ago	5 (20.8)	0.78	0.87 (0.32–2.34)		5 (20.8)	0.64	0.79 (0.29–2.13)	
Advised to test by NGO staff	22 (36.7)	0.70	0.58 (0.03–9.98)		32 (53.3)	0.14	NA	
<1 month ago	22 (37.9)				32 (55.2)			
>1 month ago	0 (0.0)	0.18	NA		0 (0.0)	0.06	NA	
Advised to test by family member/friend/other	??							
<1 month	6 (12.8)				14 (29.8)			
>1 month ago	7 (29.2)	0.09	2.81 (0.80–9.95)	2.68 (0.69–10.42)	9 (37.5)	0.51	1.41 (0.50–4.03)	

^**a**^Adjusted for gender, education, age

### Qualitative

Pathways to testing were clearly delineated by gender. Among male participants, symptoms (such as weakness, weight loss and skin problems) which were taken as a potential sign of HIV, were a common trigger for initiating the pathway for testing.

*“I had come to the skin department..I was getting small, small boils in my mouth so I had come to see the Doctor that time- I felt that I should go and do the HIV test…”* (#1269)

Among men, other triggers for testing included their wives being tested (at antenatal care or at the time of giving birth) and in one case, a wife’s presentation at hospital with symptoms of TB. For women, their husband’s illness or death due to HIV-related causes was the most common impetus for testing.

Participants reported undertaking multiple tests. For example, one male interviewee described initially being tested around 4 months after his wife tested positive for TB and HIV. He then attended GMC for testing and received a positive diagnosis but “did not take the report from them- I lost my paper”. He described not returning for testing until he started experiencing symptoms (around 6 to 7 months later). After not feeling well whilst visiting his village and subsequently being told to test for HIV by a Doctor, one male participant reported undertaking four tests with inconsistent results prior to the final positive test at GMC. This participant described his anger with Doctors when they had suggested testing and told him he was infected with HIV. At the time of recruitment to the qualitative study, he had disengaged from HIV services and was attending the ART Centre with his wife so that she could complete her CD4 count.

### The role of stigma

Internalised stigma and anticipated discrimination emerged as clear themes in participant narratives, with a distinct impact upon pathways to testing and care. Internalised stigma prevented the disclosure of HIV test results. Participants feared repercussions for themselves and their family members:

“*I did not tell anyone about this sickness. I did not speak about this sickness to anyone because I feel disgrace about it and if my co-worker come to know that I am HIV positive then they will keep distant from me*” (#1117)

Participants described their fear of meeting others from their communities whilst visiting the ART Centre, since “only those who are positive come here”. At the same time, the value of ART in enabling being able to live “normally” and being perceived to be doing so were key facilitators of accessing care:

“*I had a skin infection- big lumps all over my body. Then Doctor gave me these tablets and all the infection went. From that day I have energy for doing work and I can earn some more money. Now nobody can say I have this disease- even my company Doctor does not know it. From looking at me nobody can say I was sick.”* (#1067)

### Barriers and facilitators to attendance for HIV testing

Participants generally had a good grasp of basic facts about HIV prior to testing, eg. they understood it to be a potentially fatal condition and had an awareness of the ways in which it could (and could not) be transmitted. However, their own risk of contracting HIV was generally perceived to be low, therefore testing occurred only in the context of a secondary trigger such as symptoms or the illness or the death of a partner. For women in particular, a positive test result was often inexplicable, as their own fidelity was perceived to be protective:

*“I was so scared… why? You know I never had a sexual relationship with anyone besides my husband. My husband died.... I don’t know how he got HIV. He used to drink alcohol and stay in [another part of Goa] for work- I got it from him.” (#238)*

For some, being advised to have a HIV test (usually by a Doctor) provided motivation to attend the ICTC, with the aim of eliminating the possibility of HIV infection:

“’*why have they asked me to do the test, what have I done?’ I felt like that. But now I’ve seen things about AIDS and all, no? So I thought let me do the test and take the worry out of my mind.”* (#1440)

Two participants specifically mentioned that their awareness that testing and treatment were available free of charge was an important facilitator of attending for testing. Encouragement from healthcare workers was also reported as effective in instigating testing:

“*firstly I met one doctor is here in GMC, Dr. Anjali, she told me not to be scared whatever problem you have, you tell. I was having skin infection- my whole body had lumps, she took me inside, I was so scared but she told me ‘don’t get scared and sit inside. If something happened to you then what about your family? Who will look after them? So go down and get tested for HIV’*”.(#1067)

Participants stated they had been unable to attend for testing for economic and family-related reasons. For example, one participant reported that his work in a hotel meant that it was not possible for him to come for testing during the busy tourist season. Another stated that a lack of work meant that he did not have money to travel and had delayed his visit to the testing centre. Some participants attributed delay in their attendance for testing to sickness or death within the family, which necessitated travel or care for others.

Fear about test results was reported as a common characteristic of the testing process. Although fear relating to testing positive was cited as a motivating factor, in some cases, anxiety was clearly a barrier to accessing services:

*“I: so you thought about it and then you came* [to the ICTC] *after 4 weeks?*

*R: yes I thought of coming and then I was considering whether to go or not… go or not… and then at last I decided to come but still I came here twice and went back.*

*I: why did you come and roam around and go back?*

*R: I was feeling very different- like what would happen, if I am tested positive then what will happen in the future? Then family problem also, they will not accept me, I started thinking of all these things… that’s why I was considering whether to go for testing or not” (#1117)*

### Barriers and facilitators of accessing care

In response to a diagnosis of HIV, participants reported feelings of hopelessness, sometimes including suicidal ideation. Lack of knowledge about availability of treatment contributed to hopelessness, which led to fatalistic acceptance of their diagnosis. Among those interviewed, this was characterised as a distinct period, around the time of diagnosis which participants transitioned out of, with the help of support from NGOs, healthcare workers and family members:

“*I: when Doctor told you to do HIV testing, at that time what thoughts came in your mind?*

*R: many thoughts came in my mind… like its better off to be dead- there is no use of living now… after thinking like that I used to get a lot of tension, so my wife said ‘now its happened, what to do now? There’s no use in being tense. It is better to take medicine whilst you are alive.”* (#204)

Support was invaluable in facilitating access to care. This often took the form of advice giving and encouragement from friends, family members and healthcare workers. Services and support provided by NGOs (eg. accompanying people to appointments, guiding people through processes, providing information, particularly about treatment, provision of food rations and health insurance schemes) were cited as being particularly important:

“*First I went to […] to see one private doctor to show them my skin infection… that doctor sent me to Goa Medical College for testing and he gave me a note… I met Zindagi Goa’ people there* [at Goa Medical College] *they have helped me a lot. Whenever I feel tense Zindagi Goa always helped me.”* (#1067)

Those participants who had been very sick at the time of testing positive, recognised the role of the referral pathway between government testing centres and one of the two HIV care homes in Goa (both run by NGOs) in restoring them to health.

Needing to support and provide for family members were often important motivating factors for doing what was necessary to continue to live:

“*My wife loves me a lot and if something happens to me then who will look after them? So because of my wife and child I gained a little guts I should live for them- I am their support, only I support them, I am their main pillar. Who will look after them? Because of all these thoughts I am become a little stronger*”. (#1117)

Likewise, belief in the efficacy of medication, instilled by healthcare workers and bolstered by demonstrable impact, was an important facilitator of continuing to access care:

“*Now she, my wife, she is good* [healthy after being diagnosed with HIV and taking ART]*… Doctor told me she is good…if you take medicine you’ll be feeling good like her. Now see after taking medicine, looking at me, nobody can say I got HIV- I am fine now*”. (#1067)

## Discussion

Pathways to HIV testing were often long and complex. Triggers for testing (symptoms believed to be due to HIV, and for women, illness or death of their husband) were a response to sickness rather than awareness of risk and therefore suggestive of late presentation. Quantitative findings showed that CMD increased the possibility of delayed testing. Pathways were often characterised by a period of fear and low mood immediately before and after diagnosis, which was commonly allayed through the provision of information about treatment and support from family members/friends, healthcare and NGO workers. The reduction of symptoms associated with starting ART and the return to normal roles that this facilitated was associated with further alleviation of distress.

Worryingly, although participants were often aware of symptoms and the need for testing, this did not necessarily lead to immediate testing; participants might wait until there was another reason for visiting the hospital or until symptoms had worsened. This finding is consistent with the results of a systematic review of qualitative studies from sub-Saharan Africa which suggested that while awareness of risk provided a background motivation to test, testing was “undertaken when there was a clear decline in health which necessitates access to health care” [32]. Further evidence of poor health as a key stimulus for testing was supported by the inverse association of low scores for delayed recall with late help-seeking triggered by symptoms. A possible explanation for this finding is that it is those who are most unwell with HIV-related illnesses who perform poorly on cognitive tests [23, 33] and who therefore attend for testing without further delay.

CMD was associated with increased odds of a delay of more than 1 month between being told by Doctor or NGO worker to test for HIV and attendance for testing. This finding is consistent with our previous results that showed: a) CMD inhibited attendance for post-test counselling; b) symptoms of anxiety and depression were associated with non-attendance at the ART Centre [22]. Our results demonstrate that CMD may have an inhibitory impact earlier in the pathway testing, prior to attendance for testing. Fear of AIDS (perceived as synonymous with sickness and death) was linked to the anticipated impact of a HIV diagnosis upon ability to perform a social role (relations with families, communities, economic productivity). These concerns have been found to be a barrier to HIV testing and associated with late presentation at care [34, 35]. The impact of emotional distress around the time of diagnosis on pathways to care and HIV outcomes is unclear. The relationship between fear and distress and CMD is also uncertain. It should be noted, however, that in our study, some participants described successfully overcoming their fears and sense of hopelessness to access testing and care.

Internalised stigma and fear of discrimination formed a backdrop to qualitative narratives: inhibiting acknowledgment of risk, contributing to fear of being seen accessing HIV services and limiting access to sources of support by preventing disclosure. Evidence from elsewhere suggests a strong bi-directional link between depression and internalised stigma [36, 37]. The exact nature of the relationship between these two factors and how their interaction impacts upon engagement with care warrants further exploration.

When interpreting our findings, it is important to be aware of how the nature of our qualitative sample (those attending the ART Centre) may have influenced our findings. Although participants universally reported barriers to accessing HIV testing and care, their inclusion in our sample demonstrates that they had been, to a large extent, successful in overcoming these. Unfortunately, interviewing those who had dropped out of care was outside of the scope of the current project. Our sample may therefore be selected for resilience: our findings are likely to be skewed towards facilitators. The most vulnerable groups (those who did not collect test results, those who failed to attend the ART Centre after diagnosis) are excluded. This may explain why some factors found to be important elsewhere, ie. Financial constraints, did not feature strongly in our narratives. It will be important to carry out similar research in future with those who drop out from care. In the context of our mixed methods cohort study, interviewer resources were limited. We anticipated that a sample of 15 would enable us to get close to saturation regarding the main topics of interest. However, it is possible that a larger sample would have facilitated the emergence of additional themes. Unfortunately, validation of the pathways to care tool was outside of the scope of the Umeed study. Despite these limitations, given the dearth of evidence on pathways to HIV testing and care, our study is an important initial contribution to the evidence base.

## Conclusions

Our findings suggest that in Goa, people often delay accessing HIV testing and care until they experience undeniable symptoms, forcing them to face their fears and undergo testing. Whilst mental distress related to diagnosis may play a role in delaying access to care, this was often remedied through support from health services, NGOs, families and friends. Once contact with care and support (HIV services or NGOs) was established, these systems seemed to be effective in mitigating fear and promoting confidence in treatment and self-efficacy. The effectiveness of these systems in supporting people with mental disorder is unclear and should be explored. Screening followed by specific interventions to support people with depression to engage with care may help to shorten pathways and limit drop-out. Likewise, it is important to design research to explore the best ways to combat stigma and other barriers to early diagnosis and care, to shorten and simplify pathways to care, in order to ensure people living with HIV are able to derive maximum benefits from treatment.
